# FanFAIR: sensitive data sets semi-automatic fairness assessment

**DOI:** 10.1186/s12911-025-03184-4

**Published:** 2025-09-12

**Authors:** Chiara Gallese, Teresa Scantamburlo, Luca Manzoni, Simone Giannerini, Marco S. Nobile

**Affiliations:** 1 Department of Law, University of Turin, Lungo Dora Siena 100, 10153 Turin, Italy; 2 Department of Environmental Sciences, Informatics and Statistics, Ca’ Foscari University of Venice, Via Torino 155, 30172 Mestre, VE Italy; 3 Department of Mathematics, Informatics and Geosciences, University of Trieste, Via Edoardo Weiss 2, 34127 Trieste, Italy; 4 Department of Economics and Statistics, University of Udine, Via Tomadini 30/A, 33100 Udine, Italy; 5 Bicocca Bioinformatics, Biostatistics and Bioimaging Research Center (B4), Monza, Italy; 6 Tilburg Institute for Law, Technology, and Society (TILT), Tilburg University, Prof. Cobbenhagenlaan 221, Tilburg, 5037 The Netherlands; 7 European Centre for Living Technology, Ca′ Bottacin, Dorsoduro 3911, Calle Crosera, 30123 Venice, Italy

**Keywords:** Data bias, Fairness, Trustworthy artificial intelligence, Fuzzy logic

## Abstract

**Background:**

Research has shown how data sets convey social bias in Artificial Intelligence systems, especially those based on machine learning. A biased data set is not representative of reality and might contribute to perpetuate societal biases within the model. To tackle this problem, it is important to understand how to avoid biases, errors, and unethical practices while creating the data sets. In order to provide guidance for the use of data sets in contexts of critical decision-making, such as health decisions, we identified six fundamental data set features (balance, numerosity, unevenness, compliance, quality, incompleteness) that could affect model fairness. These features were the foundation for the FanFAIR framework.

**Results:**

We extended the FanFAIR framework for the semi-automated evaluation of fairness in data sets, by combining statistical information on data with qualitative features. In particular, we present an improved version of FanFAIR which introduces novel outlier detection capabilities working in multivariate fashion, using two state-of-the-art methods: the Empirical Cumulative-distribution Outlier Detection (ECOD) and Isolation Forest. We also introduce a novel metric for data set balance, based on an entropy measure.

**Conclusion:**

We addressed the issue of how much (un)fairness can be included in a data set used for machine learning research, focusing on classification issues. We developed a rule-based approach based on fuzzy logic that combines these characteristics into a single score and enables a semi-automatic evaluation of a data set in algorithmic fairness research. Our tool produces a detailed visual report about the fairness of the data set. We show the effectiveness of FanFAIR by applying the method on two open data sets.

## Background

Algorithmic discrimination is a problem studied in several disciplines since the nineties. The causes of discrimination are manifold and have been explored in many academic fields, such as law, ethics, sociology, and computer science. The majority of works mostly focused on biases caused by models, and produced a vast array of metrics to measure fairness in the algorithmic outputs  [1].

The term bias is used by Friedman and Nissenbaum, who argue that it “refers to computer systems that systematically and unfairly discriminate against certain individuals or groups of individuals in favor of others”  [2]. By extension, pre-existing societal bias is “bias that originates from society at large, such as from organizations (e.g., industry), institutions (e.g., legal systems), or culture at large (e.g., gender biases present in the larger society that lead to the development of educational software that overall appeals more to boys than girls)”  [3]. Pre-existing bias can be implicit and subconscious, rather than malicious in intent  [4].

One notable cause of discrimination is data bias. Scholars and activists already showed how data sets convey social bias in modern Artificial Intelligence (AI) systems, in particular those based on machine learning. For relevant examples, consider unfair outcomes in semantic representation  [5], face recognition  [6], clinical decisions  [7], speech recognition  [8], and text generation  [9] among others. Social data analysis has shown to be prone to methodological pitfalls mainly due to biases and issues related to the characteristics of the data platform and the design choices  [10]. In the machine learning community, the evaluation of fairness and bias in training and testing data sets mostly concerns transparency and documentation processes.

So far, the assessment of algorithmic fairness in the context of data sets has followed two main directions. A research track regards the analysis of data curation processes or the development of new documentation frameworks  [11, 12]. Recently,  [13] annotated hundreds of data sets employed in algorithmic research efforts specifying key fairness-related characteristics (e.g. the domain of application, the target task and the sensitive attributes encoded in the data set) in order to address the so-called “documentation debt”  [14]. Another direction focuses on the empirical analysis of fairness-related data dimensions. These efforts include pre-processing techniques such as sanitisation and instance re-weighting (for an overview see  [15]) and procedures evaluating distinct sources of discrimination in data collection  [16] or studying the effect of data-related factors on the fairness of algorithms  [17].

This work aims to advance the state of the art by providing a framework for the (semi) automated assessment of fairness in data sets. In our previous work, the problem we addressed was the following: given a data set to be used in machine learning research, how much (un)fairness can be injected by it?  [18] To answer this question we identified six fundamental features (*balance, numerosity, unevenness, compliance, quality, incompleteness*) that could influence model fairness and offer guidance in the use of data sets in contexts of critical decision-making, such as health decisions, regardless of the model used. With respect to the previous version of FanFAIR  [18], in this work we present multiple improvements, most notably: the support for two novel multivariate outlier detection method (namely Isolation Forest and Empirical Cumulative-distribution Outlier Detection) that can be used to assess the unevenness feature; an improved entropy-based balance calculation algorithm; advanced facilities for fairness reporting and plotting. The software is also now available as open-source package on PyPi.

Based on Fuzzy Logic, we elaborated a rule-based system that combines these features into a single score and allows a semi-automatic assessment of a data set in algorithmic fairness research. Our contribution is twofold: first, we identified a set of fairness indicators to support policy decisions about data use in a flexible way - i.e. allowing adaptations depending on the context of the application (e.g. the domain and the existing laws); second, we introduced a rule-based method to measure the (un)fairness induced by a data set. The fairness-related features we explore are agnostic about the sensitive attribute in that they capture essential vulnerabilities that may contribute to model (un)fairness. Another distinct aspect of our approach is to integrate statistical information about the data sets (balance, numerosity, unevenness, incompleteness) with qualitative consideration (compliance, quality) which take into account legal and ethical requirements of data-related practices, e.g. the General Data Protection Regulation (GDPR) and principles of research integrity  [19].

This integration is operated through the application of fuzzy rule-base logic and reflected in a score that can be indicative of the fairness potentially introduced by a given data set. Differently from the work of Alowairdhi and Ma, in this work we do not use fuzzy logic to assess the respect of FAIR principles (findability, accessibility, interoperability, and reusability)  [20] of a digital resource  [21], which the authors define with the word *FAIRness*, but we rather use it to quantify and integrate multiple metrics of a data set and assess the overall *fairness* of the whole resource. In fact, we focus on a different topic, integrating the ethical principles regarding fairness with those found in the law literature.

Mainstream works in computer science addresses the problem of fairness in a context of prediction-based decisions [22]. Usually, mathematical definitions requires achieving parities across distributions of outcomes or scores (common examples include statistical parity and calibration, for an overview of popular metrics see  [23]). Definitions can refer to individuals (e.g., requiring that similar individuals are treated similarly) or groups (e.g., requiring that any error measure is equal across groups, defined by so-called protected characteristics like gender or race). Mathematical definitions can reflect philosophical conceptualisations, such as the equality of outcomes and the equality of opportunities, and previous studies attempt to connect these complex, distinct literature  [24, 25]. In general, computer science literature expresses in empirical and formal terms philosophical problems of distributive justice in which the goal of fairness is to achieve an equal allocation of goods  [26].

Since there is no consensus among academics on this subject, algorithmic fairness research has led to the development of numerous mutually incompatible frameworks and formal notions of fairness in AI  [27]. Furthermore, as Birhane points out, a lot of the proposed theories only focus on technical improvements and fail to prioritise the people and communities that are disproportionately affected, while “automated and standardised solutions to complex and contingent social issues often contribute more harm than good—they often fail to grasp complex problems and provide a false sense of solution and safety. Complex social issues require historical, political, and moral awareness, and structural change.”  [28].

Along this line of research, Tubella et al. designed the ACROCPoLis framework, taking into consideration “Actors, Context, Resources, Outcome, Criteria, Power, and Links”, in order to “provide the means to represent more accurately the elements and relations that should underlie assessments of the fairness of a process and/or situation, and that can help reveal where disagreements lie when conflicting fairness assessments are defended by different parties”  [27]. Similarly, Scantamburlo et al. explored the information requirements characterising the interaction between the designer and the user of a machine learning model, which enables the implementation of algorithmic fairness  [29].

**Fig. 1:**
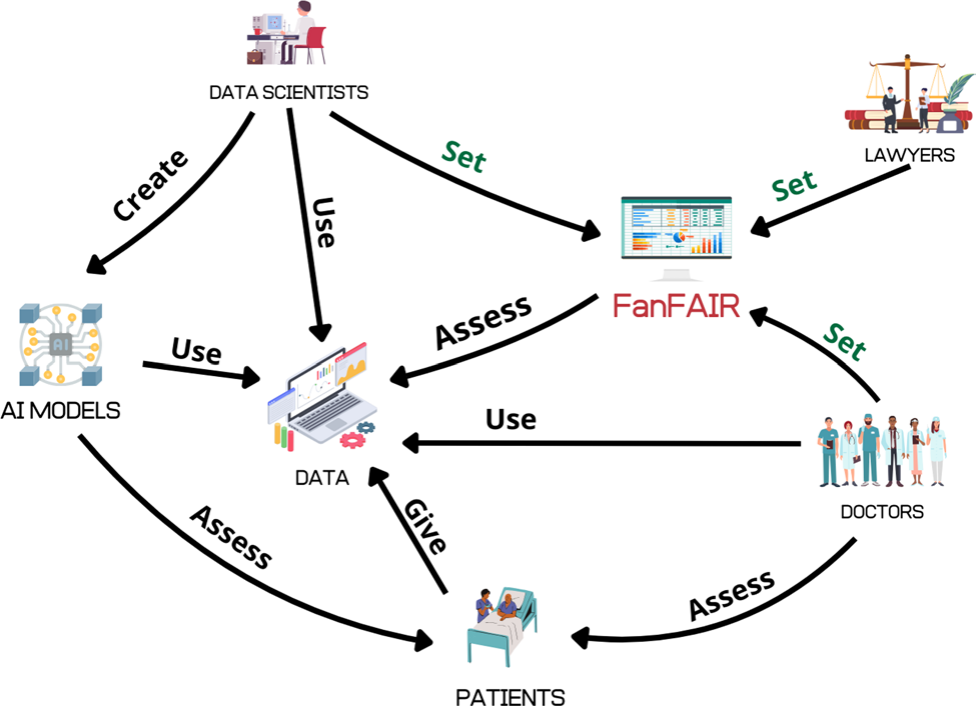
The interaction between FanFAIR, the data, and the different practitioners and stakeholders involved in their use. In general, FanFAIR can be used by data scientist, lawyers, and doctors to assess the quality of data, collected by patients and used to build AI systems

If we refer to the ACROCPoLis categories, our work identifies four different stakeholders in the ‘Actors’ category: the doctors who collect the data and assess its quality; the domain experts who perform the compliance; the AI experts who will use the data set; and the patients whose data is processed, as shown in Fig. 1. The compliance elements can be seen both under the lens of the Context and those of the Resources, while the other elements we identified fall under the ‘Criteria’ category. While ‘Power’ and ‘Outcome’ are strictly dependent on the organisational model surrounding data processing.

### Fairness in the ethics literature

In the ethics field the concept of fairness is closely tied to the concept of justice. In European and US culture, different nuances have been highlighted. Justice is usually referred to as the idea of giving each person their due, while fairness is used when talking about the capacity to judge without the interference of personal interests or bias  [30]. However, since the implications of these meanings are intertwined – being able to judge impartially often implies the reference to a standard of rightness – the two terms are often used interchangeably. Fairness has also been associated to a flexibility of judgment in contexts where a rule is too general or not applicable  [31]. For example, opting not to use an accurate machine learning model to predict the diagnosis of a subject whose characteristics are not well-represented in the training data set might be considered fair. In this case, the model may generate less accurate predictions negatively affecting the medical evaluation.

Fairness is usually ascribed to a decision impacting people and based on different criteria. For example, when a decision concerns the distribution of harms and benefits, a common guiding principle is to treat equal people equally  [32]. Note that this intuitive idea is also found in well-known formalization of algorithmic fairness  [33]. Another principle rests on the idea of grounding decisions exclusively on relevant factors or justifiable criteria, an approach that has inspired the creation of agnostic models, i.e. models which are right for the right reason  [34].

The philosophical discussion highlighted important distinctions such as the one between procedural vs. substantive justice  [35]. In the case of procedural justice the concerns revolve around the fairness of the process, while the substantive justice focuses on the fairness of the final goods allocation. Interestingly, such a distinction suggested several approaches in the algorithmic fairness scholarship such as frameworks leveraging transparency and outcome control  [36]. In contemporary philosophy, John Rawls contributed to the integration of the concepts of justice and fairness, rooting justice in a society of fairly treated people. The motivating principle behind justice is to acknowledge social interdependence and stability. According to Rawls, the cohesion of a community relies on mutual dependence among its members, and this unity can only be maintained if the institutions are perceived as just  [37].

In the field of AI, many authors have discussed the concept of fairness, as summarized by Barocas et al.  [38]; fairness is generally understood as non-discrimination, and a distinction is made between *individual fairness* and *group fairness* (also known as statistical fairness or demographic parity). Individual fairness focuses on ensuring that similar individuals receive similar outcomes from a machine learning model, while group fairness aims to ensure that different groups receive similar outcomes. Groups are usually defined by sensitive attributes such as race, gender, disability, neurotype, age, etc. The aim is that the model’s decisions should not disproportionately favor or disadvantage any particular group. However, the idea of group fairness is problematic because it does not take into consideration that multiple discriminated identities can co-exist in the same individual, leading to different AI outcomes.

### Fairness in the legal doctrine

The concept of fairness is frequently found in text of law and it has been extensively analysed by the legal scholarship  [39–41], having its roots in the “bona fides” principle in Roman law  [42] (the *bona fides* concept translates into “good faith”. In Roman law, it meant a loyal and honest behavior that would not infringe the rights of others in the execution of undertaken obligations. Good faith was the parameter used to evaluate the correctness of behaviors), and it is this concept that we are considering in the present work. However, in the context of data, and in particular health data, and in the context of AI, a legal definition of data fairness is still missing, despite being a core concept in the legal literature related to AI biases.

The new regulation on Artificial Intelligence (AI Act), which has been recently approved, represents a first step towards the regulation of data fairness (see article 10, Data Governance) since it prescribes a mandatory assessment and testing of biases in the data sets employed in AI, but it does not go so far as defining the concepts of “bias” and “fairness”, as part of the legal doctrine rejects the notion of fairness as a principle  [43, 44]. However, without a clear legal definition, fighting inequality might be very challenging due to the inhomogeneity of legal protection granted to minorities and vulnerable groups, and the practical application of the AI Act provisions remains unclear. More research is needed in this area to explore the possibility of common legal definitions at the EU level, taking into consideration intersectionality  [45, 46] and post-colonialism  [47].

Although undefined, the fairness principle is an overarching obligation in the European Digital Strategy, for example, in Art. 5, par. 1, of GDPR, and in the Data Act, but it is also found in the EU and in other countries in many pieces of law, such as Art. 8, par. 2, of the Charter of Fundamental Rights of the European Union, contract law  [48], and others  [49].

The data protection law scholarship divides the fairness principle into two elements, procedural fairness and fair balancing  [50], noting that the concept of fairness is related to the idea of vulnerability  [42]. The GDPR aims at protecting data subjects, who are in a vulnerable position as opposed to data controllers. Therefore, it tries to make a balance between the fundamental rights and freedoms of the former and the interests of the latter.

### FanFAIR’s definition of fairness

In this paper, we elaborate our own definition of fairness, which is related both with qualitative and quantitative considerations.

Although we take into consideration data protection principles, we consider several other legal principles, such as the non-discrimination principle (e.g., in the European Convention of Human Rights  [51]), the equity principle  [52], and the bona fide principle in contractual agreements  [53].

Firstly, we consider the breach of the law (e.g., copyright law, criminal law, medical law) as inherently unfair, as our fairness notion includes the lawfulness criteria, in line with the High-Level Expert Group on AI (AI HLEG)’s guidelines on Trustworthy AI  [54]. For this reason, we created the compliance feature, which includes the adherence to laws and regulations based on the respect of fundamental rights in the EU. We did not explicitly include criminal law in the definition because we assume that our tool will only be used for legitimate purposes and that the users will not engage in illegal activities.

We also included the respect of AI Ethics principles in the compliance feature, in line with the latest guidelines  [55, 56] and research on Trustworthy AI  [57].

These legal and ethical principles inspired us to include quantitative features that could facilitate the mitigation of biases from a technical point of view (see Table 1. Although the mitigation of AI biases is a complex issue that cannot be represented solely by data bias, we believe that, by improving the training data sets, AI systems will become significantly less harmful.

**Table 1:** Description of the data set features / linguistic variables considered in this work

Feature	Meaning
balance	How balanced is the data set, with respect to the output labels.
numerosity	The numerosity of instances with respect to the number of features/variables.
unevenness	The amount of outliers in the data set.
compliance	How much the data set is compliant with respect to governmental rules.
quality	A user-defined evaluation of the overall quality of the data set.
incompleteness	The proportion of missing data in the data set.

Recently, the European Medicines Agency published a document on data quality  [58], addressing some of our same concerns (e.g., extensiveness corresponds to our numerosity, and completeness features, coherence to our quality, reliability to our balance), and added other data features such as timeliness (is data available at the right time?) and relevance (is the data right for the research). In our work, we assumed that the last two concepts are part of the pre-assessment made by the data set user before employing FanFAIR: if the data is too old or unavailable, or if it is not of the right kind to be fed to the AI model to solve a given problem, then there is no point in using our software in the first place. However, we recognize that those elements are an important part of the AI life cycle and they must be included in the Data Governance assessment under the upcoming AI Act.

Qualitative considerations that cannot be automated are also important from an intersectional point of view: it is the prior evaluation of ethical principles and fundamental rights assessment in light of the data processing purpose that allows to build fair AI models. As noted by prominent literature, AI fairness cannot be fully automated  [39]. At the same time, as noted by Mittelstadt  [59], principles alone cannot guarantee fairness. The integration between automation and considerations from the social sciences perspective is the only way to mitigate inequality.

## Methods

### Fuzzy logic and simpful

**Fig. 2:**
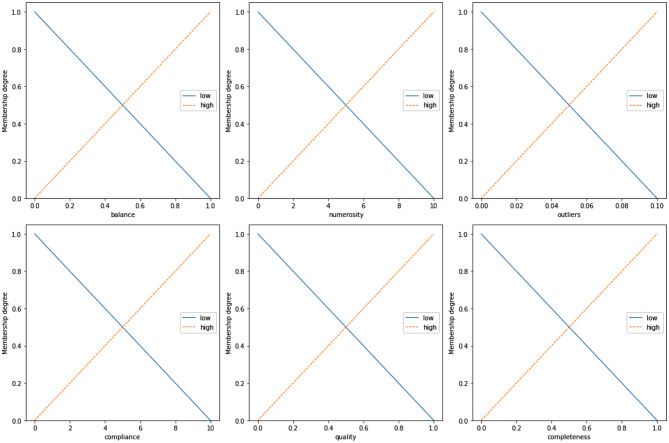
The six linguistic variables, and the associated fuzzy sets / linguistic terms, used by FanFAIR’s rule-based system

Fuzzy Logic (FL) is one form of many-valued logic that deals with approximate reasoning, in order to handle uncertain or vague concepts. FL allows computers to make partially informed decisions by taking into account imprecise, approximate, or uncertain data which gives it the ability to handle complex real-world scenarios. The concept at the core of FL is the fuzzy set, which extends traditional set theory with the possibility of elements to have a membership degree ranging from 0 (the element does not belong to the set) to 1 (the element fully belongs to the set), allowing for partial membership corresponding to intermediate values. As a consequence, in fuzzy set theory the elements can belong to multiple (possibly disjoint) sets simultaneously with different degrees of membership. In order to associate elements to membership degrees, a membership function (MF) must be defined. The simplest and most intuitive form of MF is the triangular membership, which is easy to interpret and allows for modelling the transition between fuzzy sets. Some examples are shown in Fig. 2. For instance, in the case of the “balance” linguistic variable, there are two fuzzy sets (“low” and “high”) defined using triangular membership functions. The “low” fuzzy set (in blue) is based on a triangular membership whose topmost vertex corresponds to the lowest value of “balance” (i.e., 0). This means that “0” is the element of the universe of discourse that belongs the most to the concept of “low balance”. The degree of membership to the set “low balance” decreases as we move to higher values, until it reaches zero membership in the case of element “1”, which indeed denotes a perfectly balanced data set. The same explanation, although symmetrical, could be expressed in the case of the fuzzy set “high balance” (orange fuzzy set).

For this work, we assume normalised triangular fuzzy sets (i.e., the sum of the memberships values for any element of the universe of discourse is always 1), although more complex MFs could be exploited to refine the characterisation of each variable.

Multiple fuzzy sets can be used to characterise linguistic variables, that is, variables whose values are words or sentences expressed in a natural language. For instance, a linguistic variable “room temperature” could take values “cold” or “warm”. Linguistic terms are associated to fuzzy sets that characterise the membership of any possible value in the universe of discourse to that set. Given an arbitrary temperature, we can evaluate the membership of that value to both fuzzy sets. For instance, if the room temperature is 25 degrees, the membership degree to the set “cold” will be very low, while the membership degree to the set “warm” will be quite high.

Linguistic variables and linguistic terms can be composed with logical connectors to form fuzzy rules, which are IF/THEN sentences representing implications (i.e., antecedents leading to some consequence) under specific circumstances. Specifically, a fuzzy rule takes the form:

$$\texttt{IF}\ X\ \texttt{is}\ a\ \texttt{THEN}\ Y\ \texttt{is}\ b,$$

where *X* and *Y* are linguistic variables, *a* is one of *X*’s linguistic terms, and *b* is a linguistic term for *Y* or some function used to calculate the output. Due to fuzzy sets, the antecedent can be satisfied to a certain degree; in turn, the whole rule is satisfied with a degree that ranges from 0 (not satisfied) to 1 (fully satisfied). A fuzzy inference system typically has multiple rules, covering all relevant cases. We will denote the degree of satisfaction of the *i*-th rule by $w_i \in [0,1]$. Each rule produces an output value, which is denoted by *z*_*i*_. Multiple fuzzy rules are thus evaluated and aggregated using a fuzzy inference method, which is responsible for the calculation of the final output value, according to the degree of satisfaction of each rule. In this work we exploit a 0-order Takagi-Sugeno (TS) inference system  [60], which calculates the final output of the system as follows:

1 $$output= \frac{\sum_{i=1}^N w_i z_i }{\sum_{i=1}^N w_i },$$

where *N* is the number of rules, *w*_*i*_ is the degree of satisfaction of the *i*-th rule, and *z*_*i*_ is the output constant specified in the consequent of the *i*-th rule. To build our inference system, we exploited the Simpful Python library, which is designed to simplify and streamline the building of fuzzy models  [61]. Simpful provides a set of components for the definition of fuzzy sets, linguistic variables, and fuzzy rules. In order to facilitate the definition of the rule-base – and assist the interpretation of the whole model – fuzzy rules can be entered as strings of natural text. Simpful is totally flexible, so that membership functions and fuzzy rules can have arbitrary complexity. Simpful supports multiple reasoning systems, including the 0-order TS reasoning, which was used in this work. We based FanFAIR on a TS reasoner because it is intuitive, as the consequent of a rule is a concept associated to a “crisp” number, whose semantics is straightforward to understand (i.e., low_fairness = 0, high_fairness = 100). Alternative approaches like Mamdani would use fuzzy sets in the consequents, making the interpretation of rules and results less straightforward.

We used FL for FanFAIR because it provides an intuitive method for interpreting the (un)fairness of Machine Learning (ML) datasets. As a matter of fact, given some arbitrary metric (e.g., balance) there is not a “crisp” threshold that separates a fair from a not fair data set. Thus, we cannot employ rules rooted on conventional logic rules like “if the balance is below 90% the data set is unfair”, because such a threshold is arbitrary and the huge difference between 89 and 90%is misleading. On the contrary, Fuzzy Logic gives the modeler the possibility of considering a smooth transition between the two groups, leading to a more intuitive approach to the interpretation of (un)fairness of Machine Learning datasets. By using linguistic variables, it allows us to express the degree of fairness achieved with a specific dataset in a nuanced manner, mapping the membership value on a scale from 0 to 1. These features enhance our ability to effectively communicate fairness-related information to professionals who may lack expertise in machine learning and artificial intelligence. The use of Sugeno-Takagi allows us to consider the effect of multiple rules, to give an overall assessment of the whole data set.

### Multivariate outlier detection methods

The presence of outliers in a data set (i.e., rare events or observations that deviate significantly from the rest of the data set) can be harmful in machine learning for a variety of reasons (e.g., biased parameter estimation, masking effects, loss of generalization), including privacy concerns (e.g., risk of singling-out). Because of that, abnormal values should be systematically detected, investigated and possibly fixed or removed, in case of material errors. However, Outlier Detection (OD) is a challenging tasks that can pose several challenges to the modeler. In particular, there exist an important dichotomy between univariate and multivariate OD approaches. In the former case, each variable is analyzed separately, and some score (e.g., a *z*-score) is used to determine whether a value should raise suspicion. On the contrary, the case of multivariate OD approaches is more complex as the distance between samples becomes less meaningful. Nevertheless, multivariate OD is more effective to detect outliers because it keeps into account the possible multiway dependence among variables. In this work, we improved FanFAIR by integrating two algorithms for multivariate OD (ECOD and Isolation Forest) described in the next subsections.

#### Empirical cumulative-distribution outlier detection

Empirical Cumulative-distribution Outlier Detection (ECOD) is a form of unsupervised outlier detection algorithm proposed by Li et al.  [62]. The algorithm is based on the idea that outliers are often the “rare events” appearing in the tails of a distribution.

As a first step, ECOD estimates the underlying probability distribution of the data in a non-parametric fashion by computing the marginal empirical cumulative distributions. Such distributions are then used to estimate tail probabilities. Finally, the algorithm calculates an outlier score for each data point, by aggregating the estimated tail probabilities across all dimensions. The higher the outlier score, the more abnormal that value is. ECOD uses a “contamination” hyper-parameter corresponding to the proportion of outliers in the data set. This information is used to automatically select a threshold to discriminate between outliers and inliers.

#### Isolation forest

Isolation Forest (IF) is a multivariate anomaly detection algorithm that exploits binary trees  [63]. The anomaly is estimated according to the path length needed to isolate a data point. The algorithm works by randomly selecting one feature and then selecting a split value, chosen by randomly sampling a value between the maximum and minimum values of the given feature. Given that recursive partitioning can be represented by a tree structure, the number of splittings required to isolate a sample is equivalent to the path length from the root node to the terminating node. This length can averaged over a forest of similarly created random trees. Random partitioning produces noticeably shorter paths for anomalies and outliers. Thus, when a forest of random trees collectively produce shorter path lengths for particular observations, they are highly likely to represent anomalies with respect to the rest of the data set.

## FanFAIR

In order to assess the potential fairness of a data set, we identified six different elements that should be addressed before employing the data set. Table 1 summarises the features considered for this work and gives a brief explanation of their role.

To identify each criteria, we were inspired by general principle of civil law and EU law, and established ethics guidelines  [56]. We will now provide a detailed explanation of the rationale of the features described in Table 1, along with the mathematical formalisation of the quantities chosen to measure them. In what follows, we denote by $S \in \mathbb{N}$ the sample size (number of statistical units/items in the data set). $D \in \mathbb{N}$ represents the number of features/variables in the data set.

We denote by **x**_*d*_, $d=1, \dots, D$, the vector of all values of the *d*-th variable in the data set (i.e., a “column”). $x_{s,d}$ denotes the value of the *d*-th variable of the *s*-th unit of the data set, where $s=1, \dots, S$. We use $\hat{\mu}_d$ and $\hat{\sigma}_d$ to denote the mean and standard deviation, respectively, calculated on **x**_*d*_. We denote by **y** the vector of output values (e.g., the labels).

### Balance

A data set is said to be “balanced” if all classes are well represented in the data set. The importance of balance is described in a wealth of literature: an unbalanced data set may produce biased models, unable to generalise and with prejudicial effects  [64] on single individuals or entire categories of individuals, such as disparity in accuracy  [65, 66]. For example, if a data set, used to train a model to classify healthy patients, contains thousands of patients with a disease and only a few healthy individuals, the model will probably predict the majority class and will likely be unable to recognise healthy individuals with high accuracy. The presence of imbalanced categories in classification and regression requires the adoption of appropriate statistical methods. These include using the theory of extreme/rare events or ad hoc link functions in a regression framework  [67–70]. For these reasons, it is important to be able to detect the presence of potential imbalances.

In our previous version of FanFAIR, we calculated a normalised balance by computing the relative frequency of each class in the data set. We used such information to calculate the standard deviation of the relative frequency vector: intuitively, a standard deviation of *σ* = 0 would correspond to a perfectly balanced data set; the worst case scenario is when there is only one class in the data set (i.e., the frequency is 1 for one class, and 0 for all the other classes). We denote by $\sigma^*$ the standard deviation calculated under this assumption. Therefore, $\sigma/\sigma^* \in [0,1]$ can be used to define a measure of balance of the data set as $balance = 1- \sigma/\sigma^*$, which ranges from 0 (completely unbalanced) to 1 (completely balanced). In this new version of FanFAIR, we introduce a new metric for the balance which is based on entropy. If the variable/feature has *K* categories and each feature occurs with relative frequency $\hat{p}_k$ with $k=1,\dots,K$. Then, we can use the normalized Hellinger distance as an entropic measure of deviation from the maximum balance/entropy assumption:

2 $$H = \sqrt{\frac{1- \sum_{k=1}^{K}\sqrt{\frac{\hat p_k}{K}}}{1-\sqrt{\frac{1}{K}}}}$$

The distance in Eq. 2 has been normalized to take value 0 in the case of maximum balance and 1 in the case of minimum balance, thus we redefine the new metric for the balance as:

3 $$balance= 1-H.$$

### Numerosity

This dataset feature relates to the ratio between the sample size and the number of variables/features. In the classic setup in statistics and machine learning, the sample size *S* increases whereas the number of features/variables *D* remains fixed and finite. This is the so-called fixed-dimensional framework for which a mature and sound theoretical background is available. However, many data sets in modern scientific problems have a high-dimensional character. In other words, the theoretical derivations must account for a number of features/variables *D* that increases with the sample size. Moreover, the term ultrahigh dimensional is used to indicate the case when *D* increases at a non-polynomial rate as the sample size *S* increases. Unfortunately, the classical results in probability theory and statistical inference break down in the high-dimensional case so that a new theory is required  [71, 72]. This implies that in practical applications, even if the number of features/variables and the sample size are always finite, one should identify the relevant theoretical framework that applies to their case.

A paradigmatic example of the problems arising in the high-dimensional setting is where the features and the response are independent. In such case, we would want the classification algorithm to fail to select any feature since there is no actual relation between the inputs and the outputs. Any learned model or conclusion cannot be generalized, being the byproduct of sheer chance. However, when there are more features than units in a data set, we can “fit perfectly” the training set with a linear model. This happens when linear systems of the kind $\textbf{A}x = b$, with **A** the *S* × *D* matrix of the inputs and *b* the output vector, has a solution when **A** and *b* are random. It has been shown  [73] that random linear systems with more variables (features) than equations (observations) are solvable with probability 1, i.e., even with random data it is possible to predict exactly all the output values using a linear relation. In fact, there is actually a sharp transition between almost-certain solvability and almost-certain unsolvability that depends on the actual distribution of the random data and on the ratio between the number of variables and equations  [74]. Thus, the ratio $\alpha = \frac{S}{D}$ between sample size and number of features/variables can provide information on the presence of high-dimensional data that would require appropriate statistical methods  [75].

In the above example we considered only the case of spurious linear relations, whereas in the general case, when the hypothesis space (e.g., the set of all functions representable by a specific network architecture) is known, the size of the training sample can be assessed through the Vapnik–Chervonenkis (VC) dimension, expressing the number of distinct units that can be discriminated by an hypothesis space (for an introduction see  [76], chapter seven). Hence, if the sample size is smaller than the VC dimension we can consider the sample size requirement as not satisfied since no generalisation is required and even pure noise could be learnt. The main issue when using the VC dimension is that it requires the true hypothesis space and the exact network architecture to be known and this is generally not feasible in practice.

As a first reasonable requirement, we can ask for a sample size that allows to fit a linear model in the fixed dimensionality scenario. A rule of thumb proposed in  [77] suggests that an optimal sample size *S* would be $10 \times D$. This would lead to the definition of a universe of discourse $[0,10]$ for this linguistic variable, so that the feature is calculated as:

4 $$numerosity = \min(10, \alpha).$$

Intuitively, the data set has optimal size when $\alpha \geq 10$, as shown in the corresponding membership functions of Fig. 2. Note that the requirement on the numerosity does not ensure, by itself, that for *every* model we will be unable to fit a data set where there is no relation between the inputs and the outputs. As a counterexample, consider the classical universal approximation result for multi-layer perceptrons of Hornik et al.  [78]. There, it is proved that single-layer feed-forward neural networks form a class of universal approximators. On one hand such result (and similar ones) shows that any numerosity measure is inherently flawed, since a complex-enough model can always learn perfectly even when there is no concrete relation between the inputs and the outputs. On the other hand, this shows that empirical measures that works well in practical scenarios, such the one presented here, can be considered the “best case” when there no additional knowledge on the class of functions that a certain algorithm or network architecture can learn. As a more extreme example, consider classification via *k*-Nearest Neighbors for *k* = 1. Such a classifier will learn *any* spurious relation between inputs and outputs, showing, again, that a good numerosity is not a guarantee, by itself, that only the real relations between inputs and output can be learnt, but, as with all measures, it must be considered in context, taking into account the hypothesis space given by the selected algorithm.

### Unevenness

This feature refers to the proportion of outliers in the data set. Loosely speaking, an outlier is an observation that deviates from the rest of the data (in some sense). For example, in a data set of physiological values of non-healthy patients, in the weight column we could find abnormal values (too high or too low). The domain expert will certainly understand that an adult individual cannot have a weight of either 100 grams or of 20 000 kilograms. However, outliers might sometimes be just individuals with unique features, such as a patient that weighs more than 600 kilograms. In this case, there is a risk of singling out and the patient’s identity will likely be recognised if the data set is released to the public. Clearly, the definition of outliers depends upon the assumption on the distribution of the data. A given observation could be outlying with respect to a Gaussian distribution but be compatible with other distributions, i.e. the Student’s T. Hence, besides the privacy aspects, outlying observations can produce a severe bias in modelling and classification. The field of robust statistics is active since 1960 and aims to derive methods and models that can be used when the data or the errors do not follow exactly the hypothesized distribution, for a review see  [79]. For a recent review on multivariate outlier detection methods for high-dimensional time series see  [80]. See also  [81] for a discussion on independent data.

In our previous version of FanFAIR  [18], we assumed that data is normally distributed so that we can rely on *z*-scores, a simple way to detect outliers. The *z*-score for a value $x_{s,d}$ is calculated as:

5 $$z_{s,d}=\frac {(x_{s,d}-\hat{\mu}_d)} {\hat{\sigma}_d},$$

where $x_{s,d}$ is the value of feature *d* observed on unit *s*, whereas $\hat{\mu}_d$ and $\hat{\sigma}_d$ are the mean and standard deviation of feature *d*, respectively. For simplicity, we did not exploit a Bonferroni correction and we use the heuristic to label as outliers all values such that $z_{s,d} > 3$. All *z*-scores are computed using the stats module offered by the scipy library. We then create a matrix $U \in \{0,1\}^{S \times D}$ where an element $u_{s,d}$ is calculated as follows:

6 $$ u_{s,d} = \begin{cases} 1 & \text{if}\ z_{s,d} > 3, \\0 & \text{otherwise}. \\\end{cases}$$

The total number of outliers, across all variables, was finally divided by the overall number of values in the data set to assess the Unevenness score, that is:

7 $$unenevess= \frac {\sum_{d=1}^D \sum_{s=1}^S {u_{s,d}}} {S \times D}$$

In the previous version of FanFAIR, we assumed that a data set with a ratio of outliers greater than or equal to 0.1 is already extremely uneven, so that the universe of discourse is limited to $[0, 0.1]$.

As a matter of fact, the calculation of the *z*-score can be harmful in this context, because both mean and standard deviation are strongly influenced by anomalous values. Thus, we extended the support to the modified *z*-score, where the mean is replaced by the median and the standard deviation is replaced by the Median Absolute Deviation (MAD). Moreover, in this new and improved version of FanFAIR, we also introduced the possibility of assessing uneveness by considering advanced multivariate outliers. In this case, outliers are globally detected using either ECOD or IF, as described in Sect. 2.2. The membership functions for this data set features did not change. Both ECOD and IF were implemented using the python library PyOD  [82].

### Compliance

We consider the “compliance” feature as the adherence to various legal provisions that are enforced within each country, covering relevant areas such as copyright, privacy, medical law, and others. Our framework draws inspiration from the legal principles of the European Union and civil law systems, yet it possesses the flexibility to be modified for compatibility with other legal frameworks as well. Nevertheless, we acknowledge that perceptions of fairness can vary significantly across different cultures and societies, with each community having its own unique sensitivities and expectations.

The mere act of complying with existing laws and regulations does not alone suffice to determine the ethical integrity of a dataset. However, it constitutes a critical component of such an assessment. According to the concept of Trustworthy AI set by the AI HLEG  [54], adherence to legality is a foundational pillar. Utilizing data that has been obtained through illicit means to train an AI model is unequivocally deemed unethical and unjust.

In Table 2 we present five compliance areas that we deem relevant to assess the fairness of a data set. The first area pertains to data protection laws and regulations, such as GDPR, Convention 108+, etc. In the context of fairness assessment, these principles apply even to anonymised data sets. Some important operations related to data protection are listed in Table 3.

**Table 2:** Summary of legal and ethical considerations

Area	Principles
Data Protection Law	Anonymization, Legal Basis, Data Protection Impact Assessment, Legitimate Interest Assessment, Transfer Impact Assessment, Re-use Impact Assessment, Contracts, Data Minimization, Data Retention, Data Subjects Rights, Transparency Obligations
Copyright Law	-
Medical Law	Informed Consent, Ethical Review, Clinical and Diagnostic Regulations
Non-discrimination Law	Fairness Principle
Ethics	Accountability, Dignity and Self-Determination, Traceability, involvement of stakeholders, Risk assessment, Impact on Society

**Table 3:** Data protection considerations

Term	Description
Anonymization	If the data set needs to be anonymized, the process must ensure that no re-identification is possible.
Legal Basis	The data collection and re-use must have an appropriate legal basis (in accordance with Art. 6 GDPR), e.g., consent, legitimate interest, contract, law.
Data Protection Impact Assessment (DPIA)	In the cases mentioned by the European Data Protection Board or the national Data Protection Authority, an impact assessment must be carried out (in accordance with Art. 35 GDPR).
Legitimate Interest Assessment (LIA)	In case of legitimate interest as a legal basis, a LIA must be carried out to determine if such interest exists, if it is not overridden by the interests or fundamental rights and freedoms of the data subjects, and if the “processing is necessary for the purposes of the legitimate interests pursued by the controller or by a third party” (Art. 6 GDPR).
Transfer Impact Assessment	If the data is transferred outside the EU (for example, because it is stored on a server located in a third country), a careful assessment of the impact, risks, and security implications of the transfer must be performed, as required by GDPR.
Re-use Impact Assessment (RIA)	According to GDPR, data can be reused only for purposes that are compatible with the original purposes for which the data were originally collected and processed, therefore, a careful assessment regarding compatibility must be carried out.
Contracts	In case of data transfer or joint controllership, an appropriate data agreement must be signed by the interested parties, according to the different privacy roles (Art. 28 GDPR).
Data Minimization	Only the minimum amount of data necessary to reach the processing purposes can be collected or employed (Art. 5 GDPR).
Data Retention	Data can be kept only for the minimum amount of time sufficient to fulfil the data processing purposes, and after that time it must be deleted (Art. 5 GDPR).
Data Subjects Rights	The exercise of data subjects’ rights must be ensured (Articles 12–22 GDPR).
Transparency Obligations	All transparency obligations, such as informed consent and privacy notices, must be respected, in accordance with GDPR and other applicable laws.

The second area we identified is related to intellectual property law, such as licensing. The specific applicable law and copyrights vary depending on the country, on the purposes of utilisation, and on the contractual provisions between the data owner and who is using the data. In order to be legal, both data collection and data utilisation must comply with those rules.

The third criterion is related to national laws in the medical sector, which is different in each country. Requirements regarding consent, age, reuse of data, the possibility of using deceased individuals’ data and such may vary to a great extent depending on the place in which the data is collected.

The fourth criterion is related to the non discrimination principle in national and international law and to the respect of fundamental rights of data subjects. Marginalised groups (e.g., women, foreigners, persons with a disability, neurodivergent individuals) often face discrimination in the medical practice and the collection of their data might be less accurate or less extensive, leading to a lower standard of care. In addition, the uses of the AI model trained on their data might perpetuate societal bias and have a detrimental impact on their lives. In order to ensure fairness, these circumstances need to be taken into account since the first stages of the data collection.

The fifth and last area pertains to ethical principles to be applied since the first phases of the data collection, described in Table 4.

**Table 4:** Ethical principles

Principle	Description
Accountability	This principle is present in GDPR but it is also an important ethical principle even when personal data are not involved. It means that there must be someone who takes responsibility for actions, decisions, and consequences of data collection and use.
Dignity and Self-Determination	Even when data collection and use is permitted by law, human dignity should be respected and individuals should have the right to decide how their data is used (e.g., in case of deceased individuals, their will should be respected).
Traceability	It should always be possible to trace back when, how, and by whom the ethical assessment was performed.
Involvement of Stakeholders	The persons on whom the data processing will have an impact should be heard and their opinion should be taken into account.
Risk Assessment	An assessment of ethical issues and risks should be carried out since the beginning of the data collection.
Impact on Society	The impact of the data use on society should be carefully assessed and risks should be mitigated with appropriate measures.

Due to its nature, the Compliance variable cannot be computed automatically from data, as the legal requirements may vary depending on the country of data collection and data usage, on the type of data, and, in general, on different circumstances occurring in each specific situation. The user must manually set the value of this variable by using the set_compliance method of the FanFAIR object. In particular, the user must pass as argument a dictionary where the items are the five criteria names, and the associated data are the Boolean values denoting whether that criterion is satisfied or not.

### Quality

Our definition of quality refers to evaluating the integrity of data based on expert judgment within the field, such as determining the clarity and completeness of an image, or the discernibility of sounds in a recording. The domain expert should assess the data and give an opinion on its usability for the model that will be used for the analysis.

The domain experts will discuss with the data scientists and make an assessment in relation to the problem that is intended to be solved, based on the state of the art in the specific field (such as medical imaging) and on the needs related to each specific model (e.g., the features). This evaluation is crucial as data collection is frequently conducted by various individuals without the constant oversight by data scientists (like nurses, medical interns, or students), leading to potential inaccuracies during the collection process. For example, the data collection in the clinical practice is performed most of the time for the primary purpose of providing health care, and only later might it be decided to employ such data for secondary analysis.

The quality of data also relates to the structure and implementation of the annotation framework. In very big datasets, especially those acquired through crowd-sourced platforms, the integrity of the annotations becomes a significant concern, especially in critical areas like healthcare. Quality assurance frequently includes reviewing the accuracy of annotators’ work and verifying the consistency in the labeling by different annotators. In the medical domain  [83] investigates the experience of medical experts partnering with AI practitioners to ensure data quality in image labelling tasks.

In this paper we adopt a holistic approach to data quality allowing experts to set the value of this variable manually by using the set_quality method of the FanFAIR object. The value assigned to this variable may reflect broader considerations and reasoning based on domain-specific criteria (e.g., meaningfulness, clarity of annotation instructions, transparency of annotation scheme) similar to those elaborated by fact-checking organizations  [84].

### Incompleteness

This feature addresses the issue of “gaps” (or missing values) within a dataset pertaining to critical aspects, highlighting instances where information about a subject, such as a patient’s specific health metrics, is absent. An initial approach to address these gaps involves removing the subject from the dataset if the available information is insufficient. Although straightforward, this method is less than ideal because it may result in the exclusion of valuable data. An alternative, more commonly adopted, approach is the application of imputation techniques, which employ statistical models to fill in the missing data.

There are different types of incomplete data sets:

1. some variables (e.g., dataframe columns) may have missing values. For instance, this might happen when the variable is related to some exam that is only administered to very specific patients;
2. some instances (e.g., dataframe rows) may have missing values. For instance, this might happen to fragile patients that cannot undergo some treatments or exams;
3. The missing data appears at random.

Typically, it is assumed that the mechanism that generates the missing data can be one of the following

- MCAR: Missing completely at random;
- MAR: Missing at random;
- MNAR: Missing not at random.

In detail, MCAR means that the probability of missingness is independent of the data, namely, the mechanism responsible of the missing data does not depend on the data values themselves, be they observed or missing. Under the MAR assumption, the probability of missingness is independent of the missing values, conditionally on the observed data. Typically, if the data are MAR, then non-missing data can be used to impute them. Lastly, missing data are MNAR if there is some dependence between the probability of missingness and the unobserved missing values themselves.

A key aspect of stochastic imputation methods is the preservation of the dependence structure of multivariate data. This is usually achieved by drawing observations from conditional distributions of the missing data given the observed ones (see, e.g.,  [85] and references therein).

In this work, we assume the MCAR mechanism, so that Incompleteness can be measured as the ratio between valid values and NaNs in the data set. Notice that different dataset and libraries might make different assumptions on how a missing value is represented. In particular, languages (like R) and libraries (like numpy for Python) have specific values to represent the cases when a values is missing. On the other hand, many data sets can have a missing value represented as an invalid 1 (e.g., −1 instead of a positive number) or a very large 1 (e.g., 9999 for a value that is generally below 100). Here we assume that the data set has been pre-processed and all missing values are represented as NaNs. Formally, we build an additional matrix $I \in \{0,1\}^{S \times D}$ where an element $i_{s,d}$ is calculated as follows:

8 $$ i_{s,d} = \begin{cases} 1 & \text{if NaN}, \\0 & \text{otherwise}. \\\end{cases}$$

Finally, similarly to the Unevenness score, we calculate the Incompleteness value as:

9 $$incompleteness = \frac {\sum_{d=1}^D \sum_{s=1}^S {i_{s,d}}} {S \times D}.$$

The universe of discourse of this variable ranges from 0 (all values are valid) to 1 (the data set is completely empty).

### FanFAIR’s fuzzy rule-base

The six linguistic variables described in the previous subsections are designed to capture the main sources of “unfairness” in data sets used for machine learning. A summary of the fuzzy sets, and the corresponding membership functions, is shown in Fig. 2. In order to calculate a single fairness score *φ*, that encompasses all the information about the data set, we exploit a 0-order Takagi-Sugeno fuzzy inference system  [60]. Table 5 reports the twelve rules used in this work.

**Table 5:** Rule-base used by FanFAIR for the assessment of data set fairness

Rule 1:	IF balance IS high THEN *φ* IS high_fairness
Rule 2:	IF balance IS low THEN *φ* IS low_fairness
Rule 3:	IF numerosity IS high THEN *φ* IS high_fairness
Rule 4:	IF numerosity IS low THEN *φ* IS low_fairness
Rule 5:	IF unevenness IS high THEN *φ* IS low_fairness
Rule 6:	IF unevenness IS low THEN *φ* IS high_fairness
Rule 7:	IF compliance IS high THEN *φ* IS high_fairness
Rule 8:	IF compliance IS low THEN *φ* IS low_fairness
Rule 9:	IF quality IS high THEN *φ* IS high_fairness
Rule 10:	IF quality IS low THEN *φ* IS low_fairness
Rule 11:	IF incompleteness IS high THEN *φ* IS low_fairness
Rule 12:	IF incompleteness IS low THEN *φ* IS high_fairness

The consequents’ possible output values are: low_fairness = 0, high_fairness = 100. The fuzzy reasoner was implemented using Python and the Simpful library  [61]. FanFAIR is open source and available as python package, so that it can be easily installed with pip.

## Results

In this section, we apply FanFAIR to two well-known public data sets from the UCI library: the Wisconsin breast cancer and the Parkinson data sets. These tests represent two practical use cases to investigate the feasibility of our approach. We discuss our results and the fairness estimated by our method; however, our goal is to discuss the methodology and not to assess the actual fairness of these data sets. In all tests that follow, we run FanFAIR using the following python packages: scipy v1.7.3, numpy v1.21.6, simpful v2.10.

### Use case 1: The Wisconsin breast cancer data set

The first use case that we will consider is the Wisconsin Breast Cancer data set (https://archive.ics.uci.edu/ml/datasets/breast+cancer+wisconsin+(diagnostic)) from the UCI Machine Learning Repository   [86], which describes some textural features of cell nuclei (e.g., fractal dimension, area, perimeter, smoothness) calculated on digitised images of fine needle aspirates of breast masses.

As a first step, we analysed the data set’s metadata in order to assess the compliance requirements. In the original version of this data set, the meta data reads that “Samples arrive periodically as Dr. Wolberg reports his clinical cases”. Unfortunately, no information is provided about informed consent, medical consent, nor any other elements regarding Data Protection Law and ethical review. This is not surprising, since the data set is quite old. Retrieving such information from patients would be extremely difficult, or even impossible. Thus, we assumed that the hospital correctly took care of all these aspects. Regarding copyright issues, the license is clearly stated and it allows the reuse of the data set. The overall Compliance score for this data set was then set to 4. We set the quality metrics to 70%. Finally, we selected the “ECOD” multivariate outlier detection method, and we exploited the new entropy-based balance calculation algorithm.

**Fig. 3:**
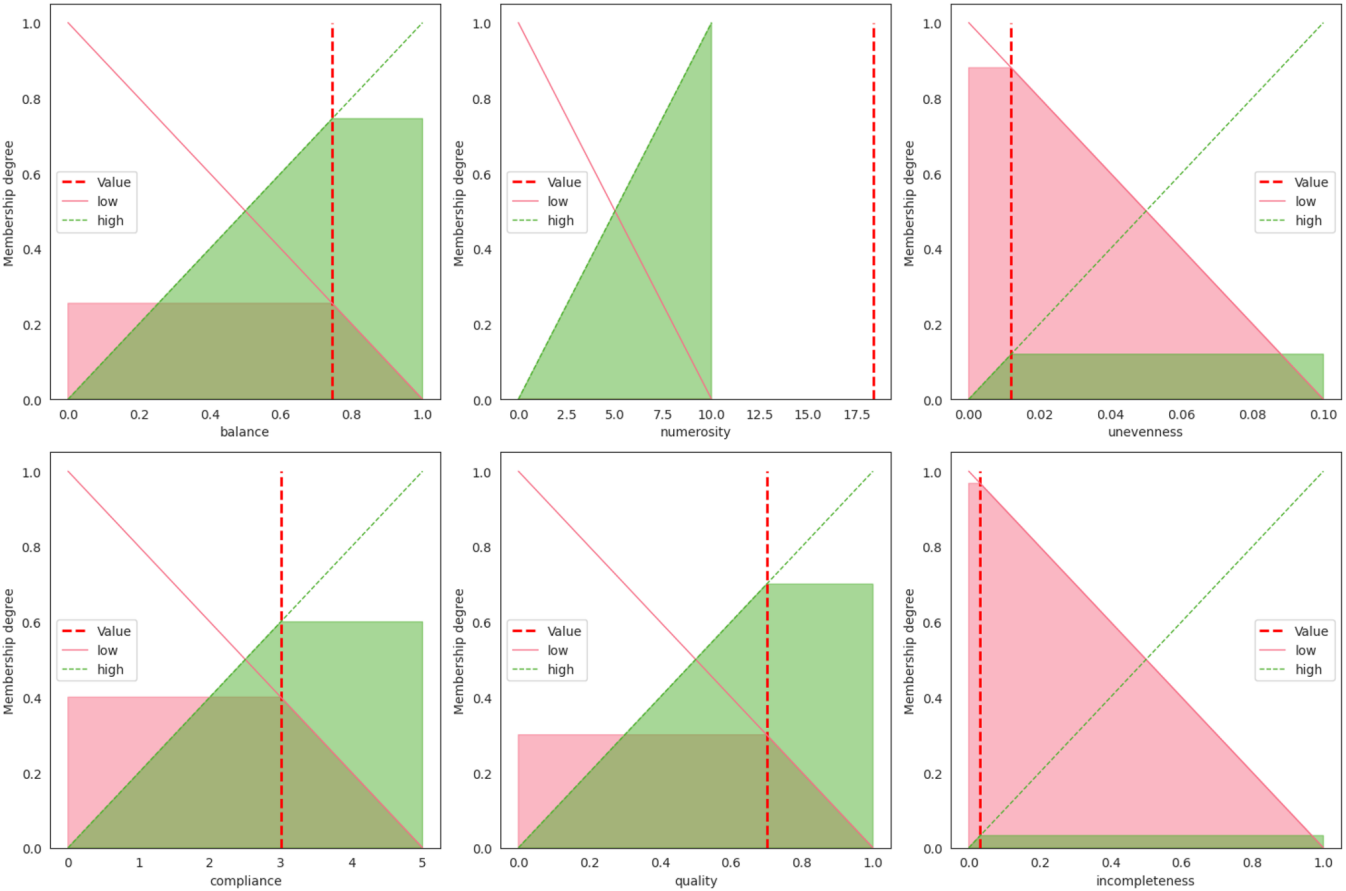
Membership values calculated for UCIs Wisconsin breast cancer data set

**Fig. 4:**
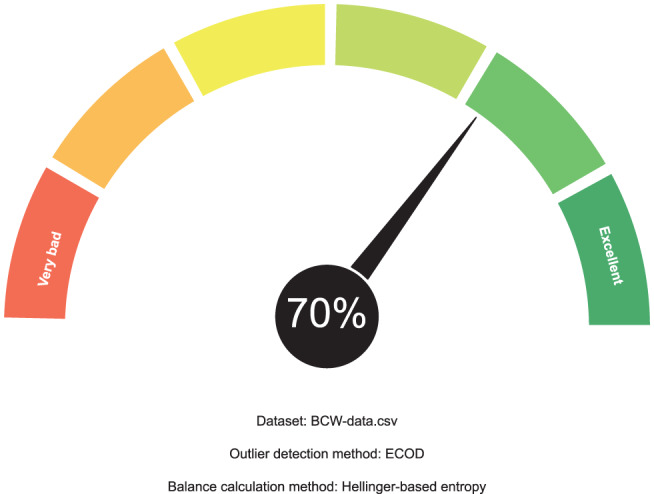
Example of summary gauge produced by FanFAIR for the BCW data set. The calculated fairness score for this data set is 70%

The output calculated by FanFAIR on this data set can be seen in Fig. 3. The first thing that can be observed is that the data set has very low incompleteness and very low unevenness. The data set is not perfectly but reasonably balanced (212 malignant vs 357 benign instances). The data set al.so has high numerosity, which was automatically capped to 10 by FanFAIR. The final fairness score calculated by the fuzzy reasoner on the Cancer data set was $\varphi=70\%$, which would make it rather suitable for research (Fig. 4).

### Use case 2: Parkinsons data set

The second use case that we tested was the Parkinsons data set (https://archive.ics.uci.edu/ml/datasets/parkinsons) from the UCI Machine Learning Repository  [87]. The data set is composed of a range of biomedical voice measurements from 31 people, where 23 suffer from Parkinson’s disease. The columns of the data set correspond to 23 specific voice measures (e.g., average vocal fundamental frequency, jittering, shimmer); the 195 rows corresponds to vocal recording from these individuals  [88]. Similarly to the previous use case, we analysed the data set’s metadata in order to find information about the data source; however, we were unsuccessful, since the data collection was not described in the data set nor in the related publication. We decided to hence give a negative score in the Ethics Compliance, while we assumed the Data Protection Law requirements were carefully respected since the paper was supported by a NIH grant. The Copyright Law has a positive score since the license allowing the reuse is clearly indicated. Overall, we assigned a 3 to the Compliance score of this data set.

**Fig. 5:**
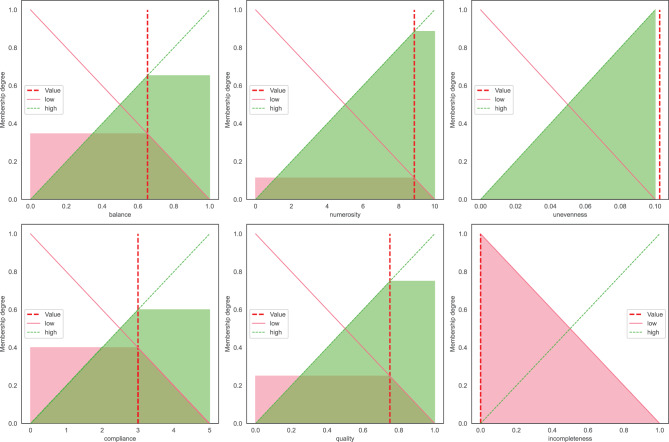
Membership values calculated for UCIs Parkinsons data set

**Fig. 6:**
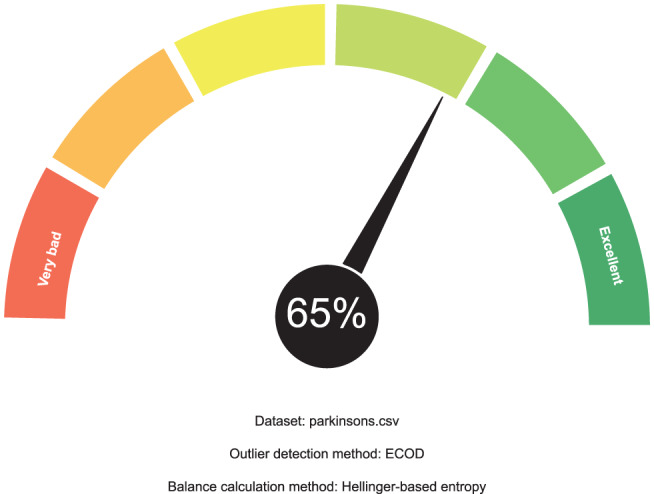
Gauge produced by FanFAIR for the parkinson data set. The calculated fairness score for this data set is 65%

According to our results, shown in Fig. 5, the data set is slightly unbalanced (entropy equal to 0.65). As a matter of fact, the data set contains 147 positive instances vs 48 negative instances. Although it is probably possible to counterbalance the circumstance by using under- or over-sampling methods, the fairness of the data set is nevertheless affected since this class of methods might introduce a slight bias in the model downstream. Finally, the data set does not have N/A values (i.e., incompleteness is zero), so that imputation is not necessary. The data set has good numerosity (8.86). Interestingly, according to ECOD, there is a 10% of outliers, causing a high level of uneveness. As a consequence, the final fairness score assessed by FanFAIR is $\varphi=65 \%$ (see Fig. 6).

One important feature of this data set is that many entries refer to the same patient. Thus, although FanFAIR calculated a high numerosity score, many instances are correlated since they come from the same patient. This circumstance can hamper the generalization, lead to a bias during the training phase and, in turn, affect the overall fairness of the data set. Unfortunately, the fact that some instances come from a same patient cannot be detected automatically from data, for it is an information that belongs to either the meta-data or is merely reported in the article associated to the data sets. We can envision the use of NLP to automatically infer similar circumstances, but that goes beyond the scope of this work.

### Using FanFAIR

We developed FanFAIR as an extremely user-friendly Python package. The tool can be installed from the PyPI repository (pip install fanfair).

Using FanFAIR is extremely simple; we show in Listing 1 a concrete example based on the Use Case 2. The first step is to import the FanFAIR object from the library (row 1). Then, the FanFAIR object is created; the constructor expects two main information: the path to the data set (specified with the argument data set, line 4), that will be internally represented as a Pandas dataframe, and the output column (line 5). The latter information is fundamental to disambiguate the input columns from the target column, which should be analysed separately to assess the the balance score. The constructor accepts two more optional arguments: drop_columns (line 6), outliers_detection_method and balance_method. The former accepts a list of column names to be dropped from the dataframe before all processing begins. The second can be used to force a specific outliers detection algorithm. The default setting is ECOD, whereas the other options are zscore, modified_zscore and IForest. The third argument can be used to specify which balance algorithm to use, whether the legacy sigma-ratio or the new version based on entropy (see, e.g., line 8).

The second mandatory step of FanFAIR is to specify the compliance scores (lines 11–15) and the quality score (line 18) for the data set. Finally, a report can be produced by calling the produce_report method, which produces the figures shown in this manuscript. It is worth noting that the report produced by FanFAIR shows both the membership values (i.e., the level of fairness with respect to each of the six criteria) and the final fairness score (which gives a final summary of the whole data set). Thus, it is possible to check which part of the data set needs to be improved (if possible) to increase the overall score. For instance, in the case of the Parkinson data set (see Fig. 5), the data set seems to be partially unbalanced: more examples of the minority class could be beneficial to produce a less biased model.

**Figure:**
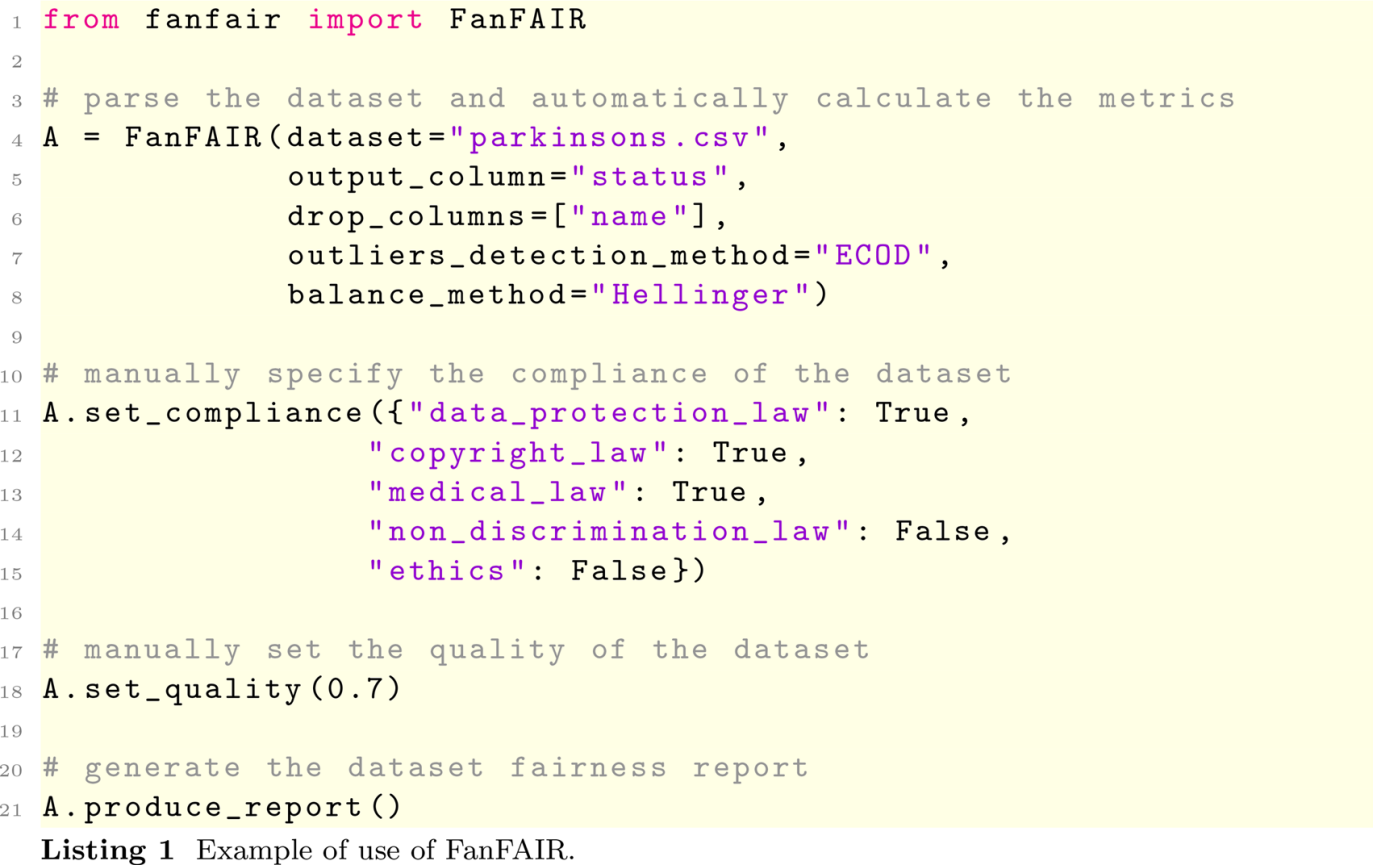

## Discussion

In the last few years we have witnessed a proliferation of metrics and technical solutions to the problem of algorithmic unfairness. The development and implementation of empirical solutions is a challenging task requiring considerations of multiple dimensions (e.g., different expertise, practical constraints vs. theoretical principles) which are poorly met by available standards. The experience with assessment of algorithms in high-stake scenarios, such as kidney transplant allocation, offers important lessons on the risk pf relegating moral choices to a purely technical sphere. As David Robinson put it “Quantification can act as a moral anesthetic. In can make morally challenging questions seem more technical and neutral than they really are.”  [89, p. 111].

The indicators provided by the FanFAIR framework cannot substitute human choices. In fact, they are meant to support policy and decision making processes suggesting relevant dimensions for the assessment of data sets used in combination with machine learning models. Scores need to be interpreted and discussed by different stakeholders, as suggested in Fig. 1. For example, some scores are strongly dependent on the statistical and numerical properties of the data sets at hand (balance, numerosity, unevenness, and incompleteness). Other two scores result from human evaluation regarding, for example, the conformity of the data sets to data protection requirements. Even though the FanFAIR software combines all indicators into a final score, finer grained evaluation can be achieved by considering indicators either individually or in groups.

The framework proposed here can be thought of as a contribution to the field of procedural fairness in that it emphasizes dimensions that are essential to manage data processing according to fairness requirement. Awareness of such dimensions, and the understanding of proper data processing, give the possibility to policy and decision makers to have realistic expectations on the fairness of data sets, and to promote meaningful data documentation as required by the AI Act and ethics guidelines.

FanFAIR is available as package on PyPI and can be installed by using the command pip install fanfair. The source code, released under the AFL 3.0 license, can be downloaded from GITHUB at the address https://github.com/aresio/FanFAIR.

## Conclusion

The widespread of AI methods across all domains (and, in particular, in high-risk sectors like healthcare) poses a serious threat to citizens’ rights and freedoms because AI systems trained on biased data sets might introduce novel biases and discriminations in society. In this paper we presented an improved version of the FanFAIR library for the assessment of data fairness, in order to provide a bias mitigation tool that can be applied even before the AI development takes place.

This new version of FanFAIR integrates novel multivariate outlier detection method, offering both Isolation Forest and the ECOD algorithms. We also provide a new improved metrics for balance based on entropy. This version was also extended with a set of functionalities for plotting and reporting, which simplify the understanding of the tool’s response.

FanFAIR is meant to serve as a guidance for doctors, data scientists, and other researchers that exploit health data for AI (in both training or data analysis) for primary or secondary use; however, our tool cannot be employed as the only instrument to mitigate AI biases, due to the multifactorial nature of such issues: the degree of discrimination or other risks to citizens does not depends solely on the dataset composition, but also on how the data is used, the type of AI model, the feature selection, the purpose of the analysis, and other factors.

With the future enforcement of the AI Act, major attention will be paid to data bias; as a matter of fact, Article 10 prescribes a complex data governance framework that includes data documentation, debasing, testing, and other requirements. As a future development, we will extend FanFAIR to also consider legal compliance tools.

In the next version of FanFAIR, will would like to introduce additional metrics and fuzzy rules to assess whether the data set is balanced with respect to any possible sensitive features (e.g., gender, age). This kind of analysis cannot be automatic and should be semi-supervised, because the user must actively specify: *1)* the sensitive features and *2)* all the possible values that each sensitive feature can assume. This implementation will require a relevant modification to FanFAIR’s interface. We also plan to extend FanFAIR to be connected to already trained ML models, in order to assess the fairness of the predictions with respect to small perturbations of the data set. Finally, we envision to integrate FanFAIR into pyFUME  [90], the python library for the development of interpretable AI systems, in order to raise an early warning to the modeler in the case of problematic data sets.

## Data Availability

FanFAIR’s source code, released under the AFL 3.0 license, can be downloaded from GITHUB at the address https://github.com/aresio/FanFAIR.

## Abbreviations

AI: Artificial Intelligence
DPIA: Data Protection Impact Assessment
ECOD: Empirical Cumulative-distribution Outlier Detection
GDPR: General Data Protection Regulation
IF: Isolation Forest
LIA: Legitimate Interest Assessment
MAD: Median Absolute Deviation
NLP: Natural Language Processing
RIA: Re-use Impact Assessment

## Declarations

### About this supplement

This article has been published as part of BMC Medical informatics and Decision Making, Volume 25 Supplement 3, 2025: IEEE CIBCB 2023 - 20th IEEE Conference on Computational Intelligence in Bioinformatics and Computational Biology. The full contents of the supplement are available at https://bmcmedinformdecismak.biomedcentral.com/articles/supplements/volume-25-supplement-3.
